# Characterization of *Aedes aegypti* Innate-Immune Pathways that Limit Chikungunya Virus Replication

**DOI:** 10.1371/journal.pntd.0002994

**Published:** 2014-07-24

**Authors:** Melanie McFarlane, Camilo Arias-Goeta, Estelle Martin, Zoe O'Hara, Aleksei Lulla, Laurence Mousson, Stephanie M. Rainey, Suzana Misbah, Esther Schnettler, Claire L. Donald, Andres Merits, Alain Kohl, Anna-Bella Failloux

**Affiliations:** 1 MRC - University of Glasgow Centre for Virus Research, Glasgow, United Kingdom; 2 Institut Pasteur, Department of Virology, Arboviruses and Insect Vectors, Paris, France; 3 Université Pierre et Marie Curie, Cellule Pasteur UPMC, Paris, France; 4 Institute of Technology, University of Tartu, Tartu, Estonia; University of California, Berkeley, United States of America

## Abstract

Replication of arboviruses in their arthropod vectors is controlled by innate immune responses. The RNA sequence-specific break down mechanism, RNA interference (RNAi), has been shown to be an important innate antiviral response in mosquitoes. In addition, immune signaling pathways have been reported to mediate arbovirus infections in mosquitoes; namely the JAK/STAT, immune deficiency (IMD) and Toll pathways. Very little is known about these pathways in response to chikungunya virus (CHIKV) infection, a mosquito-borne alphavirus (*Togaviridae*) transmitted by aedine species to humans resulting in a febrile and arthralgic disease. In this study, the contribution of several innate immune responses to control CHIKV replication was investigated. *In vitro* experiments identified the RNAi pathway as a key antiviral pathway. CHIKV was shown to repress the activity of the Toll signaling pathway *in vitro* but neither JAK/STAT, IMD nor Toll pathways were found to mediate antiviral activities. *In vivo* data further confirmed our *in vitro* identification of the vital role of RNAi in antiviral defence. Taken together these results indicate a complex interaction between CHIKV replication and mosquito innate immune responses and demonstrate similarities as well as differences in the control of alphaviruses and other arboviruses by mosquito immune pathways.

## Author Summary

Chikungunya virus (CHIKV) is a mosquito-borne human-pathogenic arbovirus of the *Togaviridae* family, genus *Alphavirus*. Arbovirus replication in vectors, such as mosquitoes, is not passively tolerated but leads to immune responses, that control virus infection. These responses therefore represent interesting targets for novel intervention strategies. Mosquito antiviral immune responses, such as small RNA pathways or immune signaling pathways, are increasingly well studied but it is not known which one mediate antiviral effects against CHIKV in particular. Here we screened four key immune responses *in vitro* for their antiviral potential against CHIKV and only the exogenous RNA interference was found to mediate antiviral activity. This was confirmed *in vivo* in *Aedes aegypti* mosquitoes. Immune signaling pathways were not found to mediate antiviral activity but were inhibited by CHIKV. This shows interesting differences and similarities to other mosquito-borne alphaviruses that increase our understanding of alphavirus-mosquito interactions.

## Introduction

Arthropod-borne viruses (arboviruses) replicate in both their vertebrate host and arthropod vector. This poses a unique problem for arboviruses as they are exposed to both the vertebrate and invertebrate immune systems. Arthropod vectors of arboviruses, such as mosquitoes, do not have the combination of innate and adaptive immune systems of vertebrates and must therefore rely on the innate immune system to control arbovirus replication. When the mosquito vector ingests a blood-meal from a viraemic vertebrate host, arboviruses infect the midgut epithelial cells. Successful replication and passage through the midgut allows the virus to disseminate into the hemocoel and infect other tissues such as the trachea, fat body, and salivary glands. Once the virus is detected in saliva, the mosquito becomes competent for transmission to the next vertebrate host [1], [2]. Therefore, interactions between the replicating virus and the mosquito immune defence system produce an outcome that can influence subsequent viral transmission, as shown for the flavivirus dengue (DENV) [3]. This emphasizes the importance of further understanding innate immunity in arbovirus vectors.

Several innate immune responses have been reported to have an antiviral effect in mosquitoes, and these include RNA interference (RNAi), as well as responses mediated by Toll, Immune Deficiency (IMD) and JAK/STAT signaling pathways [4]–[6]. The exogenous (antiviral) RNAi pathway is induced by the presence of long double-stranded RNA (dsRNA) molecules, which in the case of RNA viruses may arise from secondary RNA structures in the viral genome, the viral genome itself (in case of dsRNA viruses) and/or viral replication intermediates. Much of our understanding of arthropod RNAi is based on research on *Drosophila melanogaster*, which has also proved very useful in providing insights into antiviral responses in mosquitoes [7]. In the exogenous RNAi pathway, these dsRNAs are recognized by the RNAse III enzyme Dicer 2 (Dcr-2) [8] and cleaved into 21 nt small interfering RNA (siRNA) also known as virus induced small interfering RNAs (viRNAs) [9]–[15], a hallmark of RNAi induction. The siRNAs/viRNAs are loaded into the multi-protein RNA Induced Silencing Complex (RISC), of which the core catalytic component is the endonuclease Argonaute-2 (Ago-2) [16]. Ago-2 unwinds the siRNAs/viRNAs and retains one strand (guide strand) to recognize single-stranded (viral) complementary sequence such as genomic RNA or mRNA, which triggers the endonucleic cleavage of the complementary RNA by Ago-2 [17]. A key role for mosquito Ago-2 and Dcr-2 in antiviral responses has also been demonstrated experimentally [18], [19]. Sequence specific degradation of RNA results in repression of virus replication and virus production. Similarly, Toll, IMD and JAK/STAT signaling pathways have been described in *Drosophila* with pathway homologues also identified in mosquitoes. In the mosquito *Aedes aegypti*, the Toll and JAK/STAT signaling pathways have been shown to induce antiviral activities targeting DENV [20], [21]. In culicine mosquitoes, West Nile virus (WNV) (*Flaviviridae*) was also shown to be inhibited by the JAK/STAT pathway and that this response is thought to be controlled by the cytokine Vago [22]. In the case of alphaviruses of the *Togaviridae* family, such as Sindbis virus (SINV) or Semliki Forest virus (SFV), the data are less clear. At least in mosquito cells, antiviral activity against SFV was shown to be mediated by either IMD or JAK/STAT pathways following bacterial stimulation [23]. Recent research in *Drosophila* further implies those pathways in control of SINV infection [24], [25] although no upregulation of target genes was shown in *Ae. aegypti*-derived Aag2 cells [26]. However, in the case of *Anopheles gambiae* infection by the alphavirus o'nyong-nyong (ONNV), the contribution of the IMD pathway is only minor [27]. These pathways may therefore act in a virus-specific manner. In contrast to vertebrate cells where viral inhibition of innate immunity is an area of intense research, similar processes in arbovirus/vector interactions are poorly understood. Several arboviruses have previously been shown to inhibit or evade the antiviral action of these host responses in mosquitoes. An RNAi evasion strategy has been suggested for SFV, while flavivirus subgenomic RNA acts as an RNAi inhibitor [11], [28]. Inhibition of immune signaling pathways such as JAK/STAT, Toll and IMD has been described for SFV [23], while DENV has been shown to interfere with Toll and IMD signaling [29]. This suggests further complexity in arbovirus/vector interactions and the relevance of this is only just emerging, and needs to be assessed for each virus family and even each virus individually.

Chikungunya virus (CHIKV) belongs to the genus *Alphavirus* of the family *Togaviridae*. It has become an increasingly important arbovirus in tropical and subtropical regions, resulting in febrile and arthralgic disease in humans [30]. After the large outbreak in the Indian Ocean in 2004, CHIKV infections have spread throughout the Indian Ocean and Africa into Southern Europe [31]. Moreover, the virus has emerged for the first time in the Americas (St. Martin/Martinique, Caribbean) in December 2013, thus potentially threatening other parts of the New World (see WHO Website for information). CHIKV is spread by aedine mosquitoes with *Ae. aegypti* being the most important vector and more recently *Ae. albopictus* following changes in the E1 envelope protein [32], [33]. The viral genome is a single stranded positive sense RNA containing two open reading frames: one expressing the non-structural polyprotein which will be cleaved into non-structural viral proteins (nsP1-4), and one structural polyprotein which will be cleaved into the structural proteins. The mRNA encoding the structural polyprotein is transcribed from the subgenomic promoter during virus replication. Little is known about innate immune responses induced by CHIKV during infection of mosquitoes. Identification of small RNAs derived from CHIKV in aedine mosquitoes and their derived cell lines proves the ability of the RNAi pathway to target CHIKV. Moreover, 21 nt viRNAs as well as another class of virus-derived small RNAs with characteristics of Piwi-interacting RNAs (piRNAs) were discovered [12]. However, nothing is known about the functional ability of this RNAi response to limit CHIKV replication in mosquito cells or mosquitoes, or the involvement of other innate immune responses.

In this study, we investigated the major antiviral *Ae. aegypti* immune pathways and assayed their ability to control CHIKV replication in cell culture, where experimental conditions are easily controlled and manipulated. The exogenous RNAi pathway was identified as a key control mechanism of CHIKV replication and this was further confirmed in mosquitoes. Moreover, CHIKV repressed Toll, IMD and JAK/STAT pathway stimulation *in vitro* by induction of host cell shut off and none of the pathways were able to mediate antiviral responses against CHIKV replication. These data indicate the importance of RNAi as a mosquito antiviral response also targeting CHIKV replication as well a complex interplay with other host responses.

## Methods

### Viruses and cell culture

CHIKV E1-226V strain (provided by the French National Reference Center for Arboviruses, Institut Pasteur) was used for mosquito infections. The strain was isolated from a patient on La Reunion Island in November 2005 as part of a survey during an outbreak, as described in [34].

Vero cells were cultured in DMEM (Dulbecco's Modified Eagle Medium) supplemented with 10% fetal bovine serum (FBS), 1000 units/ml penicillin, 1 mg/ml streptomycin, and maintained at 37°C and 5% CO_2_. *Ae. aegypti* Aag2 and *Ae. albopictus* C6/36 cells (for virus propagation) were grown in Leibovitz L-15 insect medium with 10% FBS, 10% tryptose phosphate broth, 1000 units/ml penicillin, and 1 mg/ml streptomycin at 28°C.

For CHIKV titer determination, Vero cells were grown to confluent monolayers in 6-well plates, infected with 10-fold serial dilutions of virus for 1 h, and then overlaid with an agarose-nutrient mixture. After 3 days incubation at 37°C, cells were stained with a solution of crystal violet (0.2% in 10% formaldehyde and 20% ethanol). The total number of plaques was counted and the titer was calculated. Titers are indicated as plaque forming units (PFU)/ml.

### Plasmids and CHIKV-derived replicons

Use of plasmids pAct-*Renilla* (coding sequence of *Renilla* luciferase (RLuc) under control of the *Drosophila* actin 5C promoter), p6×2DRAF-Luc (coding sequence of firefly luciferase (FFLuc) under control of a multimerised *Drosophila* STAT-responsive element), pJL195 (coding sequence of FFLuc under control of the *Drosophila attacin A* promoter) and pJM648 (coding sequence of FFLuc under control of the *Drosophila drosomycin* promoter) in signaling pathway experiments have been previously described [23]. Luciferase SP6 Control DNA plasmid (Promega) expressing FFLuc under control of SP6 promoter was used as a template for *in vitro* transcription of a control RNA.

Nonstructural open reading frame of CHIKVRep(3F)RLuc-SG-FFLuc replicon contains RLuc encoding sequence inserted between codons for amino acid residues 383 and 384 of CHIKV nsP3. In place of the structural open reading frame, mRNA encoding for FFLuc is transcribed following replication by use of the subgenomic promoter. Replicon CHIKVRep-SG-eGFP contains the nonstructural open reading frame without any reporter genes and mRNA of eGFP is transcribed following replication by use of the subgenomic promoter.

### *In vitro* transcription

Replicon DNA was linearized by digestion with *Not*I, purified using a PCR purification kit (Roche) and 1 µg of the linearized DNA was *in vitro* transcribed using MEGAscript SP6 polymerase kit (Ambion) in the presence of Cap analog (Ambion). Control Luciferase SP6 DNA was linearized by digestion with *XmnI*, purified by gel extraction and *in vitro* transcribed as before. For all subsequent experiments, 2 µl of *in vitro* transcription was transfected.

### dsRNA production for mosquito transfection

Total RNA was extracted from adult *Ae. aegypti* using the RNA II Nucleospin kit (Macherey-Nagel GmbH & Co.) according to the manufacturer's instructions. cDNAs were generated from 60 ng of total RNA by reverse transcription using SuperScript II Reverse Transcriptase (Invitrogen) and oligo dT. To synthesize dsRNA, cDNA was amplified with gene specific primers (table 1 designated as being for use *in vivo*) incorporating the T7 RNA polymerase promoter sequences (in bold) at the 5′ ends. Primers were designed to amplify a unique ∼500 bp region in the PIWI domain for Ago-2. PCR was carried out using the KOD Hot Start DNA Polymerase (Novagen). PCR products were purified with the QIAquick Gel Extraction kit (Qiagen) and dsRNA was produced using the MEGAscript RNAi kit (Ambion) according to the manufacturer's instructions.

**Table 1:** Table of all primer sequences.

Primer	Sequence 5′-3′	Use
Ago-1 F	**GTA ATA CGA CTC ACT ATA GGG** ACA GGT TTC ACT GTT CAA CCT	dsRNA (*in vitro*)
Ago-1 R	**GTA ATA CGA CTC ACT ATA GGG** GGT TTG ACC GTT TTC TAG CTG C	dsRNA (*in vitro*)
Ago-2 F	**GTA ATA CGA CTC ACT ATA GGG** GCC CTC AAC AAG AAA CAC C	dsRNA (*in vitro*)
Ago-2 R	**GTA ATA CGA CTC ACT ATA GGG** GGC GTT GAT CTT GAG CCA	dsRNA (*in vitro*)
eGFP F	**GTA ATA CGA CTC ACT ATA GGG** GGC GTG CAG TGC TTC AGC CGC	dsRNA (*in vitro*)
eGFP R	**GTA ATA CGA CTC ACT ATA GGG** GTG GTT GTC GGG CAG CAG CAC	dsRNA (*in vitro*)
Ago-2 F	**TAA TAC GAC TCA CTA TAG G** CA GTT CAA GCA GAC GAA CCA	dsRNA (*in vivo*)
Ago-2 R	**TAA TAC GAC TCA CTA TAG G** TG ATG TAG ACG CGT CCT CTG	dsRNA (*in vivo*)
Luc F	**TAA TAC GAC TCA CTA TAG G** GC GCC CTG GTT CCT GGA AC	dsRNA (*in vivo*)
Luc R	**TAA TAC GAC TCA CTA TAG** **G**GA GAA TCT CAC GCA GGC AGT TC	dsRNA (*in vivo*)
Ago-2 F	GGC TGC TCA CCC AAT GTA TCA AGA	qRT-PCR
Ago-2 R	AAC CGT TCG TTT TGG CGT TGA T	qRT-PCR
Ago-1 F	GTA CGA TGC GTC GTA AGT AC	qRT-PCR
Ago-1 R	GTA CTT GTC GAG GAA GTA TTT GG	qRT-PCR
S7 F	CCA GGC TAT CCT GGA GTT G	qRT-PCR
S7 R	GAC GTG CTT GCC GGA GAA C	qRT-PCR

All primer sequences are shown reading from 5′ to 3′. Bases in bold indicate the T7 promoter sequence. The use of each primer pair is also indicated.

### dsRNA production for transfection of cultured mosquito cells

RNA was extracted from Aag2 cells using TRIzol and reverse transcribed using Superscript III Reverse Transcriptase (Invitrogen) following the manufacturer's instructions. PCR products were generated with T7 promoter sequences at either end of the fragment using the primers listed in table 1 and designated as for use *in vitro*. The PCR product was blunt end cloned into a pJet1.2 vector (Thermo Scientific) following the manufacturer's instructions. Cloned PCR fragments were verified by sequencing. PCR was performed on the cloned fragments and the products purified using the PCR purification kit (Roche). peGFP-C1 (Clontech) was used as a template for the amplification of control dsRNA, targeting eGFP. dsRNA was synthesized using the Megascript RNAi kit (Ambion) following manufacturer's instructions.

### Transfection and signaling pathway stimulation experiments

24 h prior to transfection, 1.7×10^5^ Aag2 cells were seeded in 24-well plates to reach 70% confluence the following day. For knockdown experiments, 500 ng of dsRNAs were transfected into Aag2 cells using Opti-MEM and Lipofectamine2000 (Invitrogen) according to manufacturer's instructions. At 24 h post dsRNA transfection, cells were transfected with capped *in vitro* transcribed replicon RNA derived from CHIKVRep(3F)RLuc-SG-FFLuc using Lipofectamine2000. Cells were lysed in 1× passive lysis buffer (Promega) 24 h post replicon transfection and luciferase activities determined.

For the signaling pathway stimulation experiments, bacterial stocks were prepared by inoculation of 1 µl *E. coli* JM109 in 5 ml L-Broth and incubation at 37°C for 16 h or 5 µl *B. subtilis* inoculated in 5 ml L-Broth incubated at 37°C for 8 h. *E. coli* is used to stimulate the JAK/STAT and IMD pathways and *B. subtilis* is used to stimulate the Toll pathway. Cultures were centrifuged at 1174 g at 4°C for 10 minutes. The bacterial pellet was then resuspended in 500 µl PBS and heat inactivated at 80°C for 10 minutes. At 24 h post seeding, Aag2 cells were transfected with 12.5 ng pAct-*Renilla*, 25 ng p6×2DRAF-Luc, 25 ng pJL195 or 500 ng pJM648 and capped *in vitro* transcribed CHIKV replicon RNA (derived from CHIKVRep-SG-eGFP) at 24 h post seeding, using Lipofectamine2000 according to the manufacturer's protocol. Cells were stimulated at 24 h post transfection with heat inactivated *E. coli* JM109 or *B. subtilis* for 1 hour at 28°C. 12 h post stimulation, cells were lysed in 1× passive lysis buffer and luciferase activities determined.

### Mosquitoes and intrathoracic inoculation of dsRNAs

*Ae. aegypti* Liverpool strain (provided by D. Severson, University of Notre Dame, IN, USA) were maintained on 10% sugar solution at 28°C with a photophase of 16 h and 80% relative humidity according to the standard rearing procedures.

RNAi-based gene-silencing assays were conducted by injecting 500 ng of dsRNA (dsAgo-2 or dsFFLuc) in water into the thorax of cold-anesthetized 4 day-old females using a Nanoject II microinjector (Drummond Scientific Company). Blood feeding was carried out 48 h post dsRNA injection. Gene silencing validations were performed at day 1, 2, 3, 4 and 7 after ingestion of the infectious blood-meal. As controls, mosquitoes injected with PBS and non-injected unfed mosquitoes were used.

### CHIKV infections of mosquitoes

Adult female mosquitoes were deprived of sugar source 24 h before infection and allowed to feed on artificial blood-meals consisting of a virus suspension (1/3 vol/vol), washed rabbit erythrocytes (2/3 vol/vol), and 5 mM ATP. The artificial blood-meal was provided in glass feeders covered with a chicken skin membrane and maintained at 37°C. Females placed in plastic boxes were allowed to feed for 15 minutes. Engorged females were selected, transferred into cardboard containers, provided with sugar solution and maintained in BSL-3 insectaries until analysis. To determine the titer of the infectious blood-meal which would be used for subsequent RNAi-based gene-silencing assays, three different virus titers: 10^6^, 10^7^, 10^8^ PFU/ml were tested. At indicated time points, 10 females were tested for the presence of CHIKV by immunofluorescence assay (IFA) on head squashes using mouse ascitic fluid raised against the virus [35]. Disseminated infection rates (DIR) corresponding to the number of females with disseminated infection among tested females, were determined. At various days post infection, mosquitoes were analyzed as follows: midgut and head were isolated from each individual and ground in 150 µl DMEM before being homogenised and filtered. 50 µl of the filtrate was titrated by plaque assay on Vero cells to estimate the number of infectious viral particles. The remaining 100 µl was used for quantification of Ago-2 and ribosomal S7 RNA by real time quantitative RT-PCR.

### Total RNA extraction, real time quantitative RT-PCR, and PCR

Dissected mosquito organs (midgut and head) were homogenized separately and used for RNA extraction using the NucleoSpin 96 RNA kit (Macherey-Nagel GmbH & Co). An equal amount of RNA extracted from each organ was examined for each time point. Quantification was carried out by real time quantitative RT-PCR using the Power SYBR Green RNA-to-CT one step kit (Applied Biosystem).

Total RNA was extracted from cultured cells using TRIzol (Invitrogen) according to the manufacturer's instructions. Polyadenylated RNAs were reverse transcribed using the Superscript III kit (Invitrogen) and oligo dT primers (Promega) according to the manufacturer's recommendation. Quantification was carried out by real time quantitative PCR using the Fast SYBR green master mix (Invitrogen).

All PCR reactions were done in triplicate. Specificity of the PCR reactions was assessed by analysis of melting curves for each data point. Values were normalized against the *Aedes aegypti* ribosomal protein S7 gene. Following real-time quantitative PCR assays, analysis of relative expression of Ago-1, Ago-2 and S7 was performed according to the 2^−ΔΔCt^ method [36].

### Luciferase assay

Luciferase expression was determined using the Dual Luciferase kit (Promega). Luciferase activities were determined on a Glomax Multi+ Microplate Multimode reader (Promega).

### Statistical analysis

Virus titer means were compared using the Kruskall-Wallis test from the STATA software (StataCorp LP). Statistical significance for replicon replication after knockdown was determined by a paired Student's t-test and statistical significance in the stimulation experiments was determined using a general linear mixed model.

## Results

### Effects of RNAi effector knockdowns on CHIKV replication in Aag2 cells

**Figure 1:**
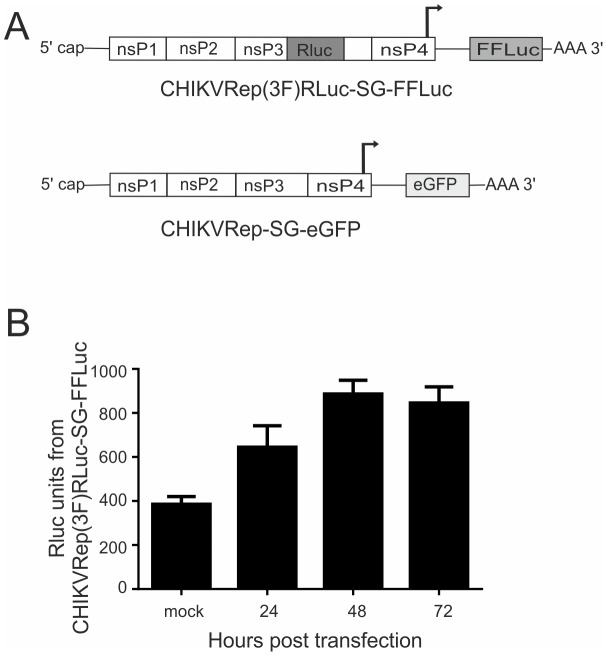
Replication of CHIKVRep(3F)RLuc-SG-FFLuc in Aag2 cells. (**A**) Schematic representation of CHIKV replicons. RLuc was inserted into the C-terminal region of the nsP3 protein and sequence encoding for FFLuc was cloned in place of the structural open reading frame under the control of the subgenomic promoter to give CHIKVRep(3F)RLuc-SG-FFLuc. A second replicon with no reporter in the non-structural open reading frame and coding sequence of eGFP cloned under control of the subgenomic promoter was created, CHIKVRep-SG-eGFP. (**B**) Aag2 cells were transfected with *in vitro* transcribed CHIKVRep(3F)RLuc-SG-FFLuc RNA and RLuc activity measured 24, 48 or 72 h post transfection (hpt). Graph shows the mean RLuc readings from a representative single experiment carried out in triplicate. Error bars show the standard error of mean. Three independent experiments have been carried out in triplicate.

Aag2 cells have proven to be a reliable model for the study of aedine immune responses including those against viruses given the presence of all major immune signaling pathways and small RNA pathways in the cell line [10], [11], [14], [26], [29], [37]. Not only have mosquito cell lines been shown to have intact immune pathways but they also provide a robust system where experiments can be performed in a controlled manner and have been used extensively in the mosquito immunity field [10]–[12], [23], [29], [38]–[40]. Viral replicons are useful tools to investigate innate immune responses in a tightly controlled manner in cell culture experiments. We first engineered two CHIKV reporter replicons on the basis of the CHIKV E1-226V strain. The first replicon, named CHIKVRep(3F)RLuc-SG-FFLuc, encodes a non-structural polyprotein with RLuc inserted into the C-terminal region of nsP3 and mRNA of FFLuc expressed from the viral subgenomic promoter instead of the mRNA of structural proteins. A second replicon is expressing mRNA of eGFP from the viral subgenomic promoter: CHIKVRep-SG-eGFP (Fig. 1A). This replicon was designed with a fluorescent protein as a reporter rather than luciferase to allow a multitude of different experiments to be performed.

To determine if these replicons are functional in mosquito cells and to characterize their kinetics with regards to the expression of the reporter proteins, Aag2 cells were transfected with *in vitro* transcribed capped CHIKVRep(3F)RLuc-SG-FFLuc, lysed at different time points post transfection (24, 48 and 72 hours post transfection; hpt) and luciferase expression determined. Luciferase expression (both FFLuc [data not shown] and RLuc) can be measured by 24 hpt with a peak at 48 hpt and a slight decrease at 72 hpt (Fig. 1B). These time points were chosen to allow sufficient time for replicon replication but also taking into consideration the transient nature of transfections. These data suggest that the engineered replicons are replicating in Aag2 cells.

**Figure 2:**
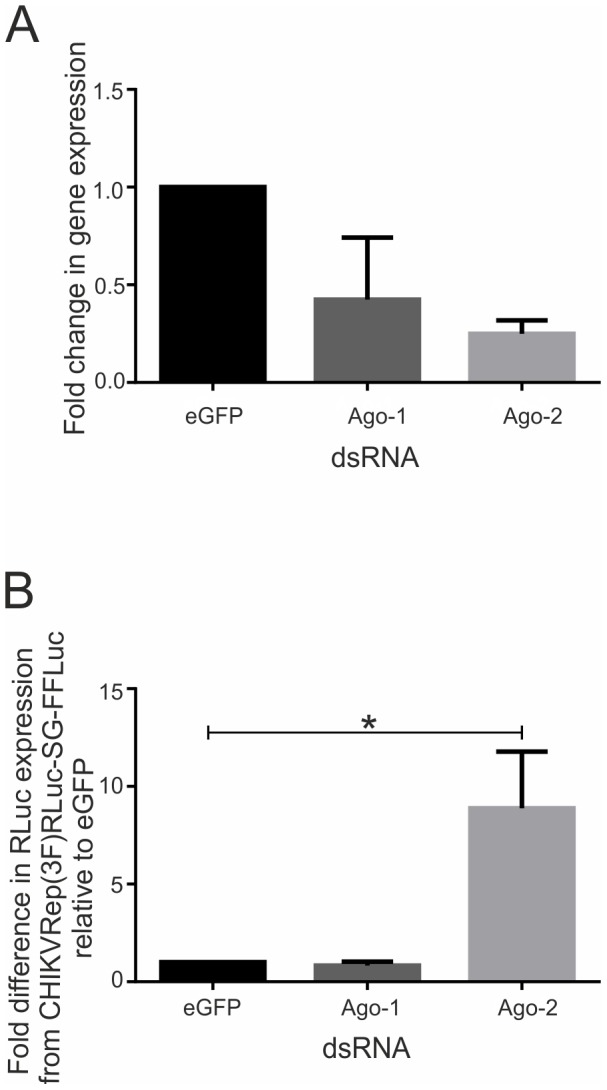
Knockdown of Ago-1 and Ago-2 in Aag2 cells and effect on CHIKV replication in Aag2 cells. (**A**) *Ae. aegypti*-derived Aag2 cells were transfected with 500 ng dsRNA and target transcripts analysed by qRT-PCR. Graph shows the relative transcript expression relative to eGFP-specific dsRNA treated cells and normalised to S7. Error bars show standard error of mean. (**B**) Aag2 cells were transfected with 500 ng of indicated dsRNAs, followed by transfection of *in vitro* transcribed CHIKVRep(3F)RLuc-SG-FFLuc replicon RNA and RLuc levels were measured. Graph shows mean RLuc levels from three independent experiments performed in triplicate. Error bars show the standard error of mean. *, Student's t-test, p = 0.045.

The RNAi pathway has been identified as being the major antiviral pathway in control of replication of a number of arboviruses in mosquitoes [4], [5]. The generally accepted method of confirming antiviral activity in mosquito cell culture and mosquitoes is RNAi mediated knockdown of components of individual small RNA pathways [3], [18], [19], [37]. Therefore, in order to determine whether the exogenous RNAi pathway also limits CHIKV replication in Aag2 cells, unique dsRNAs were designed and validated against the exogenous RNAi pathway component Ago-2 and the miRNA pathway component Ago-1 [4], [5]. Knockdown was determined by quantitative RT-PCR (Fig. 2A). Having shown efficient knockdown of both Ago-1 and Ago-2 (42% or 25% respectively), their contribution in control of CHIKV replication was assessed. Aag2 cells were transfected with dsRNA (Ago-1 and Ago-2 specific or eGFP-specific as a control), followed by transfection of *in vitro* transcribed capped CHIKVRep(3F)RLuc-SG-FFLuc and lysed after 24 hpt of replicon. A significant (Student's t-test; p = 0.045) 9-fold increase in RLuc expression was observed in cells treated with Ago-2 specific dsRNA compared to cells with control dsRNA. In contrast, no significant increase in RLuc expression was observed for cells transfected with Ago-1 specific dsRNA (Fig. 2B). These data suggest that the miRNA pathway is not involved in the inhibition of CHIKV replication in *Ae. aegypti*-derived cells, in contrast to the exogenous RNAi response that is able to inhibit CHIKV replication *in vitro*.

### Effects of Ago-2 dsRNA injection on CHIKV infection and dissemination in *Ae. aegypti*

Having shown an inhibitory effect of the exogenous RNAi pathway on CHIKV replication in mosquito cells, similar experiments were performed in mosquitoes to determine if *Ae. aegypti* RNAi components are required for defence against CHIKV *in vivo*.

**Figure 3:**
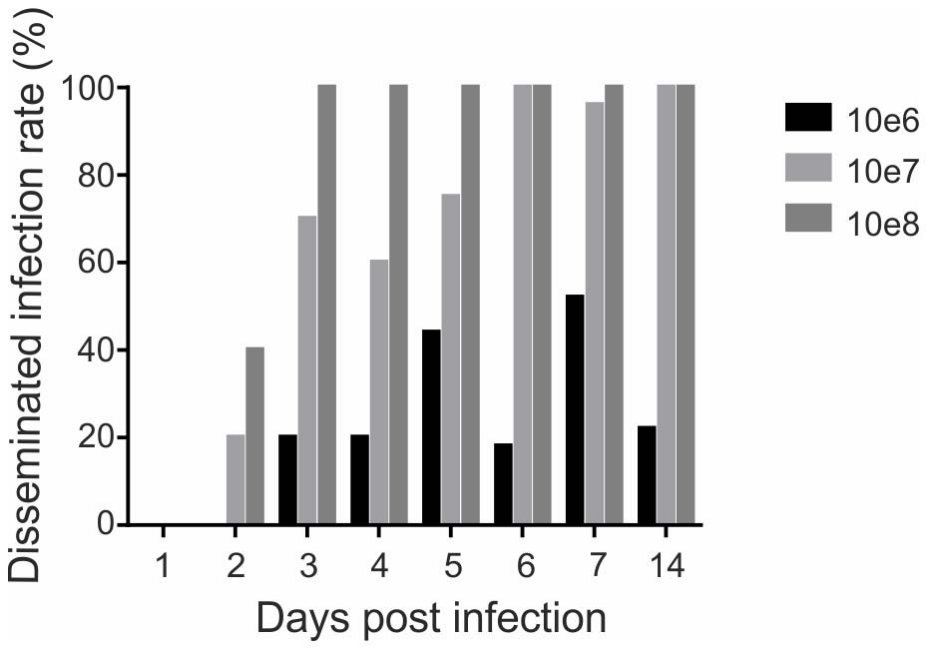
CHIKV infection in *Ae. aegypti*. Disseminated infection rates (DIR) were determined by estimating the proportion of mosquitoes with disseminated infection (where infection has spread beyond the midgut and led to infection of secondary organs including the salivary glands) among tested females. Three viral titers were tested: 10^6^, 10^7^ and 10^8^ PFU/ml.

**Figure 4:**
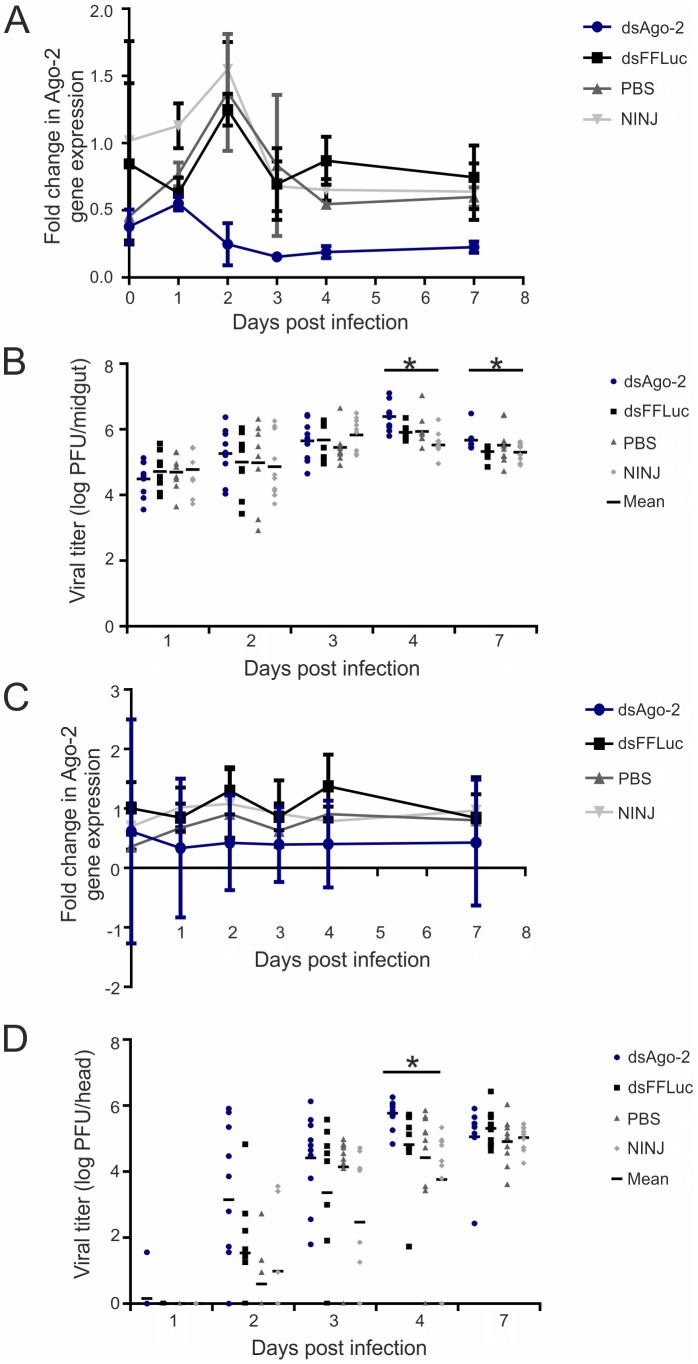
Infectious viral particles in midguts and heads of *Ae. aegypti* mosquitoes injected with Ago-2-specific dsRNA and infected with CHIKV. Two days after injection of 500-meal at a titer of 10^7^ PFU/ml. The expression of Ago-2 in midguts (A) and heads (C) was calculated relative to the expression of internal control S7 gene at each day post infection (pi) by real-time quantitative PCR. At indicated days pi, PFUs as measure of virus replication were determined by plaque assay of (B) midguts and (D) heads. 10 individuals were analyzed at each day pi. Controls were FFluc-specific dsRNA-injected, PBS-injected and non-injected (NINJ) mosquitoes. Bars indicate standard deviations. Significance (*) was determined by using Kruskall-Wallis test (p<0.05).

First, CHIKV infection kinetics was determined in *Ae. aegypti* mosquitoes, infected with bloodmeals containing different virus titers, by subsequent determination of dissemination rates by IFA on head squashes at several time points (Fig. 3). Maximum dissemination was observed at day 7 post infection (pi) with 52% for a blood-meal titer of 10^6^ PFU/ml, at day 6 pi (100%) for 10^7^ PFU/ml, and at day 3 pi (100%) for 10^8^ PFU/ml. Based on these patterns, the intermediate titer of 10^7^ PFU/ml was chosen for further assays on RNAi-based gene-silencing. Gene silencing efficiency was then tested. 500 ng dsRNA (Ago-2 specific or FFLuc specific as a control) was injected into mosquitoes 48 h prior to infectious blood-meal (CHIKV at 10^7^ PFU/ml). Ago-2 expression (relative to S7 expression) in midguts or heads of injected mosquitoes was determined by real-time quantitative PCR at time points indicated (1, 2, 3, 4 and 7 days pi). The S7 gene was chosen as a control housekeeping gene due to its stability in infected and non-infected conditions [data not shown]. Mosquitoes were analyzed at days 0, 1, 2, 3, 4 and 7 pi. Injection of Ago-2 specific dsRNA strongly decreased the expression of Ago-2 in midguts (Fig. 4A: ranging from 80% silencing at day 2 to 70% silencing at day 7) and heads (Fig. 4C: ranging from 68% at day 2 to 50% at day 7) compared to the controls (FFLuc specific dsRNA dsFFLuc, PBS or non-injected mosquitoes). Silencing was effective from day 2 up to 7 pi.

To test whether silencing of Ago-2 expression would increase CHIKV replication in *Ae. aegypti* following an infectious blood-meal, midguts and heads of the previous experiments were examined for CHIKV production. The number of infectious viral particles was determined by plaque assay of midguts and heads of 10 females at day 1, 2, 3, 4, and 7 pi (Fig. 4 B and D). A non-significant increase of virus in midguts was observed between mosquitoes injected with Ago-2 specific dsRNA and controls (dsFFLuc, PBS and non-injected) at day 1, 2 and 3 pi (Kruskall-Wallis Test: p>0.05). However, at day 4 and 7 pi a significant increase in the number of virus particles in midguts was detected following Ago-2 knockdown (Kruskall-Wallis test: p<0.05) (Fig. 4B). To determine the effect of gene silencing on viral dissemination, the number of viral particles in heads was also determined. At day 4 pi, heads of mosquitoes injected with Ago-2-specific dsRNA contained significantly more infectious viral particles (10^3.1^±10^2.3^) (Fig. 4D) than controls (Kruskall-Wallis test: p<0.05). This effect was transitory as at day 7 pi, viral loads remained similar when compared to other treatments. Taken together the *in vitro* and *in vivo* data imply a control of virus replication by the exogenous RNAi pathway with the virus being unable or not efficiently able to avoid this antiviral response.

### Viral inhibition of innate immune pathways

**Figure 5:**
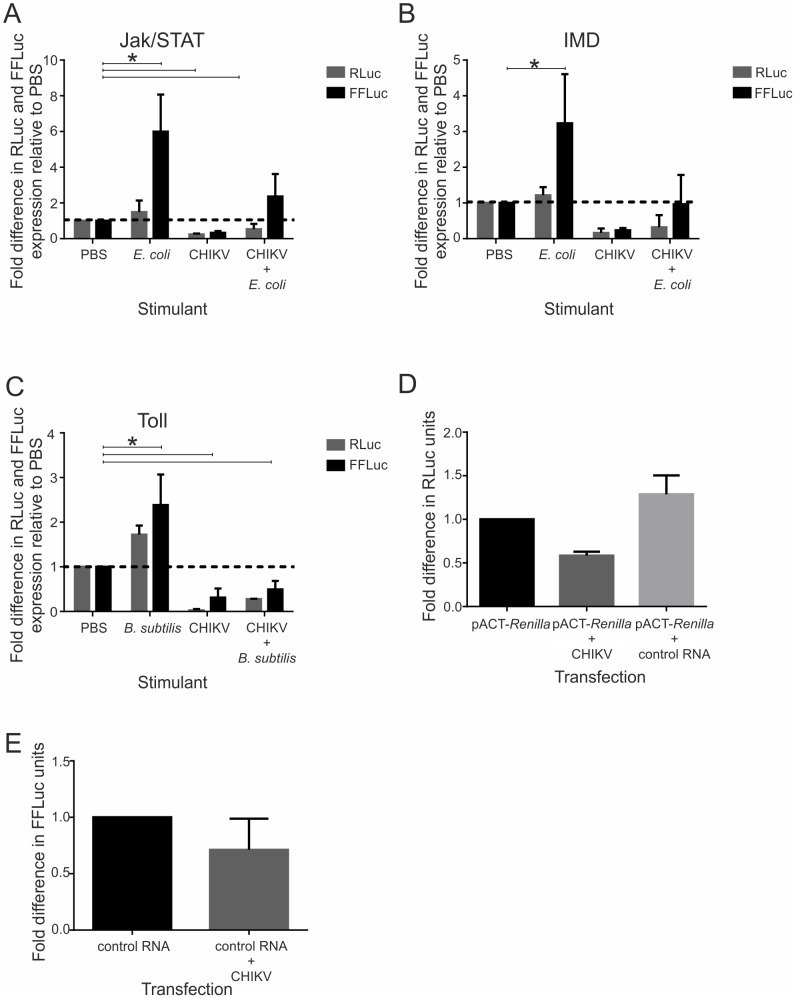
CHIKV inhibition of JAK/STAT, IMD and Toll signaling pathways. Signaling assays were performed as previously described [23]. Aag2 cells were co-transfected with reporter plasmids and *in vitro* transcribed CHIKVRep-SG-eGFP RNA, followed by stimulation of immune pathways by heat inactivated bacteria. Signaling activity was measured by activity of FFluc. Internal transfection control (RLuc) is shown to indicate the levels of gene expression. PBS, PBS added to cells containing only the pathway reporter and internal transfection control; *E. coli*, heat inactivated bacteria added to cells containing only the pathway reporter and internal transfection control; *B. subtilis*, heat inactivated bacteria added to cells containing only the pathway reporter and internal transfection control; CHIKV, PBS added to cells containing pathway reporter, internal transfection control and CHIKVRep-SG-eGFP replicon RNA; CHIKV+*E. coli*, heat inactivated *E. coli* added to cells containing pathway reporter, internal transfection control and CHIKVRep-SG-eGFP replicon; CHIKV+*B. subtilis*, heat inactivated *B. subtilis* added to cells containing pathway reporter, internal transfection control and CHIKVRep-SG-eGFP replicon. Data is shown after subtraction of background luciferase levels and relative to PBS control. (**A**) JAK/STAT pathway stimulation with heat inactivated *E. coli* in the presence or absence of CHIKV replicon. (**B**) IMD pathway stimulation with heat inactivated *E. coli* in the presence or absence of CHIKV replicon. (**C**) Toll pathway stimulation with heat inactivated *B. subtilis* in the presence or absence of CHIKV replicon. (**D**) Co-transfection of pACT-*Renilla* with either CHIKVRep-SG-eGFP replicon RNA or control FFLuc expressing RNA. (**E**) Co-transfection of control RNA in the presence and absence of CHIKVRep-SG-eGFP replicon RNA. Graphs represent the mean of three independent experiments performed in triplicate and error bars represent the standard error of mean. *, significantly different using a general linear mixed model.

Similar to other arboviruses, CHIKV may have evolved mechanisms which allow the virus to evade or suppress the induction of innate immune signaling pathways [6]. To investigate this possibility, signaling assays were performed in Aag2 cells similar to those described previously [23]. First, inhibition of innate immune pathways by CHIKV RNA was determined. Gene expression studies have shown the presence of JAK/STAT, IMD and Toll in Aag2 cells, which make them suitable models for the subsequent experiments [26], [29]. Preliminary experiments indicated the suitability of reporter gene expression studies and bacterial stimulation to study these pathways also in our Aag2 cells [not shown]. Therefore, Aag2 cells were co-transfected with *in vitro* transcribed capped CHIKVRep-SG-eGFP (not expressing luciferase) and plasmids encoding FFLuc under control of promoters that are activated in response to immune signaling of the JAK/STAT (p6×2DRAF-Luc), IMD (pJL195) and Toll (pJM648) pathways and an internal transfection control plasmid pAct-*Renilla*. This was followed by stimulation of the different pathways by either heat inactivated *E. coli* or *B. subtilis* and expression of FFLuc and RLuc was detected. Stimulated cells without CHIKV replicon showed a significant increase in FFLuc expression in case of *E. coli* or *B. subtilis* respectively for both JAK/STAT, IMD and Toll stimulations (Fig. 5 A–C control versus *E. coli* or *B. subtilis* [p = <0.001]). Cells co-transfected with the CHIKV RNA and pathway specific constructs, showed a much reduced level of stimulation compared to cells lacking the CHIKV replicon after IMD stimulation and a highly significant reduction in the level of stimulation after JAK/STAT and Toll stimulation (2.5 fold less JAK/STAT stimulation, 3.4 fold less IMD stimulation and 4.9 fold less Toll pathway stimulation in the presence of CHIKV) (Fig. 5 A–C control versus CHIKV+*E. coli* or CHIKV+*B. subtilis* [p = <0.001]). However, presence of the CHIKV replicon RNA also significantly reduced RLuc expression, regardless of which pathway was tested, compared to cells lacking the CHIKV replicon (Fig. 5 A–C control vs CHIKV [p = <0.001]). Moreover, inclusion of the CHIKV replicon RNA reduced the RLuc expression levels without stimulation of other pathways (Fig. 5 A–C control versus CHIKV [p = <0.001]). The reduction in RLuc expression is specifically due to the presence of the CHIKV replicon as co-transfection of pACT-*Renilla* plasmid and a control luciferase RNA expressing FFLuc did not result in a reduction of RLuc expression (Fig. 5D). These data suggest that, similar to findings for the related alphavirus SFV [23], CHIKV can shut down, albeit incompletely, the host cell transcription/translation systems and this general targeting mechanism also interferes with immune pathway stimulation, especially with regards to the Toll pathway which cannot be stimulated in the presence of CHIKV. In order to determine on which cellular process CHIKV is exerting its effect, CHIKV replicon RNA was co-transfected with control FFLuc expressing RNA and the translation of FFLuc assessed by luciferase assay. While there is a small reduction in FFLuc expression upon co-transfection of CHIKVRep-SG-eGFP replicon, there is not the same significant effect on the control RNA in the presence of CHIKV (Fig. 5E) as there is during the co-transfection with the pACT-*Renilla* plasmid. Therefore, similar to the related SINV and SFV, it is likely that CHIKV host cell shut-off occurs at a transcriptional level [41]–[43]. It should be noted that this incomplete or weak shut-off does not appear to have the detrimental effects often observed in alphavirus-infected vertebrate cells, but nonetheless affects host cell signaling.

### Control of CHIKV replication by JAK/STAT, IMD and Toll signaling pathways

**Figure 6:**
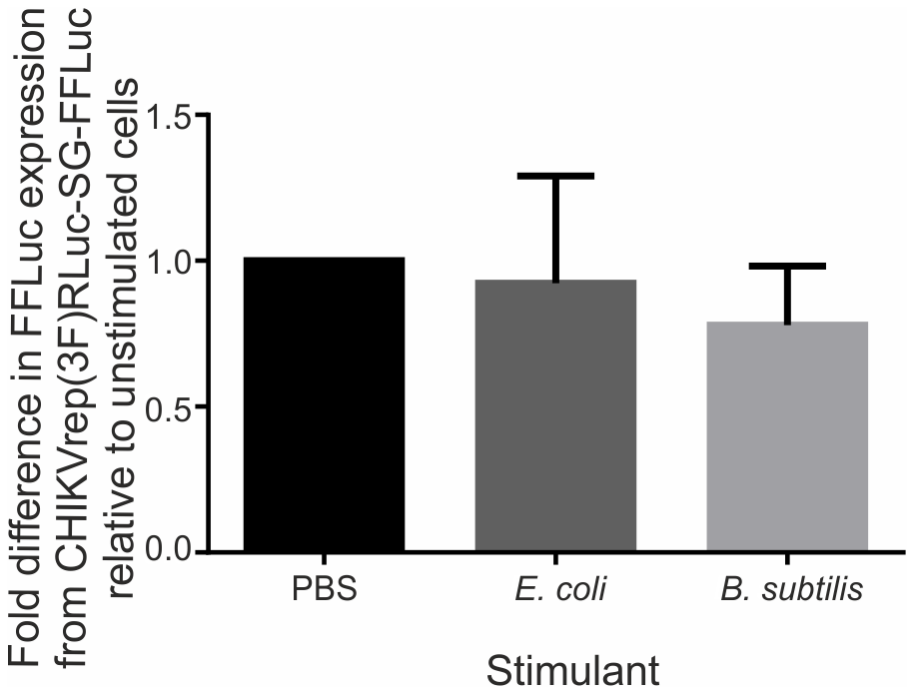
Effects of pathway stimulation on CHIKVRep(3F)RLuc-SG-FFLuc replication. Aag2 cells were pre-stimulated with heat inactivated bacteria, followed by transfection of CHIKVRep(3F)RLuc-SG-FFLuc replicon RNA. Replicon replication was assessed 12 h post pathway stimulation by FFLuc activity. PBS, PBS added to cells containing CHIKVRep(3F)RLuc-SG-FFLuc replicon; *E. coli*, heat inactivated bacteria added to cells containing CHIKVRep(3F)RLuc-SG-FFLuc replicon; *B. subtilis*, heat inactivated bacteria added to cells containing CHIKVRep(3F)RLuc-SG-FFLuc replicon. The dark grey bar shows CHIKVRep(3F)RLuc-SG-FFLuc replication after JAK/STAT and IMD pathway stimulation with heat inactivated *E. coli*. Light grey bar shows CHIKVRep(3F)RLuc-SG-FFLuc replication after Toll pathway stimulation with heat inactivated *B. subtilis*. Replicon replication after bacterial stimulation is calculated relative to replicon replication after stimulation with PBS (black bar). The graph represents the mean of three independent experiments performed in triplicate and error bars represent the standard error of mean.

As CHIKV was shown to inhibit immune signaling via host cell shut off, we hypothesized these pathways could induce antiviral activities. In order to investigate these possibilities, Aag2 cells were stimulated with either heat inactivated *E. coli* (to induce JAK/STAT and IMD signaling) or *B. subtilis* (to induce Toll signaling) prior to transfection of *in vitro* transcribed and capped CHIKVRep(3F)RLuc-SG-FFLuc. Replication was determined by measuring by FFLuc expression (Fig. 6). Stimulation of cells prior to replicon transfection had no effect on CHIKV replication regardless of whether cells were stimulated with gram negative or gram positive bacteria. This suggests the Toll, IMD and JAK/STAT pathways cannot affect CHIKV replicon replication.

## Discussion

Innate immune responses are important for regulating arboviral replication in mosquitoes. The JAK/STAT, IMD and Toll immune signaling pathways have been individually implicated in the control of arbovirus replication in mosquitoes in a virus/pathway-dependent manner [20], [21], [23], [25]. In addition, the exogenous RNAi pathway is a key mosquito antiviral pathway [4], [5]. The re-emerging CHIKV is known to induce an RNAi response in infected mosquitoes [12]. CHIKV infection of mosquitoes and their derived cell lines results in the production of small RNAs indicating that small RNA pathways are activate against CHIKV and that the presence of an RNAi inhibitor resulted in increased virus replication and virus production [12]. This study, however, did not identify which small RNA pathways have antiviral activity. Expression of the RNAi inhibitor B2 by CHIKV affected both exogenous RNAi and piRNA pathways thus making it unclear which of these pathways affected viral replication. Indeed, our study identifies one such pathway specifically. Here we have demonstrated that Ago-2 plays an important role in the antiviral RNAi response to CHIKV both *in vitro* and *in vivo*, and that the induced exogenous RNAi response mediates effective antiviral activities. The exogenous RNAi pathway has previously been shown to mediate antiviral activities against three other alphaviruses, SINV, SFV and ONNV [18], [19], [37]. In the case of CHIKV infection, production of small RNAs produced by the exogenous RNAi and piRNA pathways has also been demonstrated [12] and our data demonstrate that the exogenous RNAi does indeed mediate antiviral activity. In the case of SINV infection of *Ae. aegypti* mosquitoes, knockdown of Ago-2 resulted in a transient increase in virus replication and titer early in infection; however, similar to our findings, the effect was lost by day 7 [18]. Additionally, engineering the Flock House Virus RNAi inhibitor B2 protein into SINV increased the mortality rate, indicating that RNAi is vital in controlling virus replication to levels that are non-pathogenic to the arthropod vector [44]. A similar effect was reported for exogenous RNAi control of DENV replication in *Ae. aegypti* [3]. Our results extend the importance of the exogenous RNAi pathway to another virus of medical importance, CHIKV. Knockdown results for Ago-2 directly show that CHIKV replication is inhibited by the RNAi pathway and that the small RNAs produced in the exogenous RNAi pathway do mediate antiviral activity. It has been postulated that arboviruses may in fact subject themselves to RNAi control to ensure vector survival, which may explain why no RNA silencing suppressor (RSS) proteins have been identified to date in arboviruses. Recently, an RSS was found in the flaviviruses DENV and WNV. The RSS was not a viral protein but a highly structured region in the 3′ UTR of the flavivirus genome called subgenomic flavivirus RNA (sfRNA) which is proposed to act as an RNAi inhibitor [28]. It is yet to be seen if a similar mechanism is employed by alphaviruses to escape replication control by RNAi although an evasion mechanism has been suggested for SFV [11].

Antiviral responses other than RNAi in mosquitoes are beginning to emerge, and these innate immune pathways play a vital role in antiviral defence in a virus dependent manner. We have identified that in particular the Toll immune signaling pathway is strongly inhibited by CHIKV *in vitro*, most likely by a mechanism involving host shut-off. The JAK/STAT and IMD pathways have previously been reported to be involved in anti-viral defense against SINV in *Drosophila* [24], [25]. On the other hand, gene array studies suggested inhibition of Toll signaling late in infection and possibly activation of IMD signaling in SINV-infected *Ae. aegypti* mosquitoes [45]. However, in Aag2 cells infected with SINV gene expression studies revealed no activation of these pathways (with the exception of weak upregulation of STAT itself) [26]. The design of these experiments with SINV makes it difficult to come to any conclusion with regards to viral inhibition, but recent work has shown that SFV as well as DENV have been able to inhibit innate immune signaling in mosquito cells [23], [29]. Despite this, IMD and/or JAK/STAT but not Toll can mediate antiviral activity against SFV, while Toll and JAK/STAT induce antiviral responses against DENV [20], [21], [23]. We found no effect of any of these pathways on CHIKV replicon replication either because they do not act antivirally or because viral inhibition of any antiviral effects is strong enough to mask these. This points to virus specific differences or highly efficient inhibition of the immune pathways by CHIKV. Interestingly, only a minor activity by the IMD pathway was shown against ONNV [27], which is closely related to CHIKV therefore these viruses may respond in a more similar manner. Overall, this interplay between antiviral signaling and viral inhibition is strongly reminiscent of viral interactions with the innate immune responses, such as the interferon system of vertebrate cells [46]–[48]. At least for SFV, and our observations with CHIKV, shut off of host gene expression appears to mediate signaling inhibition in mosquito cells, as it does in vertebrate cells [23]. The mechanism by which shut-off occurs is not clear, although our data (Fig. 5E) suggest an effect prior to translation, possibly at the transcriptional level as occurs in vertebrate cells [41]–. We cannot rule out competition between host and viral mRNAs, differences in mRNA stability or other processes that would lead to differences in translation, however, as reporter mRNAs are not affected by CHIKV replicon this appears unlikely. Other inhibitory strategies employed by arboviruses may also resemble those used in vertebrate cells. In the presence of RNAi, virus replication may still be controlled to a non-harmful level. Recent work has indicated candidates for antimicrobial peptides, attC and dptB, that inhibit SINV replication in *Drosophila* [24]. Similar mechanisms in mosquito cells remain to be determined. Clearly, there is no direct effect of these classical immune signaling pathways on CHIKV replication under our experimental conditions.

In conclusion, we demonstrated that the exogenous RNAi pathway plays a vital role in limiting CHIKV replication in cell culture and in mosquitoes. Knockdown of Ago-2 resulted in a significant increase in RNA replication and virus titers. Additionally, we have shown that CHIKV significantly represses Toll pathway stimulation, and stimulation of major insect immune pathways did not limit CHIKV replication. This indicates that antiviral immunity is a complex process which needs more research to tease out the complexities of the virus/host interactions and differences exist even between closely related alphaviruses. Exogenous RNAi is also active against CHIKV while analysis of immune signaling pathways indicates differences to other arboviruses including other alphaviruses. Taken together this and other studies suggest that the RNAi response is the most generally active antiviral pathway in mosquitoes.
